# The Current Approach to Diagnosis and Management of Left Ventricular Noncompaction Cardiomyopathy: Review of the Literature

**DOI:** 10.1155/2016/5172308

**Published:** 2016-01-11

**Authors:** Courtney E. Bennett, Ronald Freudenberger

**Affiliations:** ^1^Mayo Clinic, 200 First Street SW, Rochester, MN 55902, USA; ^2^Lehigh Valley Health Network, 1250 S Cedar Crest Boulevard, Allentown, PA 18103, USA

## Abstract

Isolated left ventricular noncompaction (LVNC) is a genetic cardiomyopathy characterized by prominent ventricular trabeculations and deep intertrabecular recesses, or sinusoids, in communication with the left ventricular cavity. The low prevalence of patients with this cardiomyopathy presents a unique challenge for large, prospective trials to assess its pathogenesis, management, and outcomes. In this paper we review the embryology and genetics of LVNC, the diagnostic approach, and propose a management approach based on the current literature available.

## 1. Introduction

Isolated left ventricular noncompaction (LVNC) is a genetic cardiomyopathy characterized by prominent ventricular trabeculations and deep intertrabecular recesses, or sinusoids, in communication with the left ventricular cavity [1]. The clinical sequelae of these deformities are the syndrome of heart failure and the risk for arrhythmias and stroke. Dusek first described the postnatal persistence of spongy myocardium in 1975 pathologically, but Engberding and Bender made the first clinical recognition with two-dimensional (2D) echocardiography in 1984 [2, 3]. Three decades later, with only morphologic assessment available and no definitive genetic pathway, isolated left ventricular noncompaction (LVNC) remains a diagnostic and management challenge. In this review, we wish to define a unified process for diagnosis and suggest a management approach with special attention to guidance for anticoagulation and prevention of sudden cardiac death (SCD).

## 2. Embryology

The pathologic theory of LVNC is the failure of compaction during fetal development. Cardiomyocytes form a tube in the midline of the embryo from the mesodermal primordia and differentiate into myocardium based on multiple genetic factors, including positive and negative gene regulators [4]. Ventricular myocardial trabeculations are evident in the human heart by the end of the first trimester and occur as protrusions from the endocardial layer. The trabeculations allow for a greater surface to volume ratio and increasing muscle mass before the establishment of the coronary arteries. The next step includes compaction of the trabecular layers. This begins in the human embryo by 10 to 12 weeks and by the fourth month of gestation the compacted myocardium composes the majority of the ventricular volume [4, 5]. Compaction continues into the postnatal period with continued growth and increasing systemic pressures. The final process is development of the spiral pattern of the myocardial fibers, which is responsible for the twisting nature of contraction. Without the completion of compaction, there is myocardial dysfunction secondary to the failure of the efficient rotational ventricular system to develop for contractile performance. This concept has been demonstrated by abnormal speckle tracking and is further discussed later [6].

## 3. Genetics

There are multiple genetic proposals for the phenotypic development of noncompaction [1, 7–12]. None of them have been consistently identified to be the single gene abnormality causing LVNC, but they are briefly included here for completeness (see the list below) [7]. It is established that a thorough, three-generational family history should be obtained for evaluation of genetic influence, which may also impact the screening of additional family members [1, 7, 8, 10]. A systematic review by Bhatia et al. identified a familial occurrence rate of 30% in family members that were screened based on an index case [13]. Oechslin and Jenni have proposed an acquired pathogenesis in patients with prior normal cardiac structure and function that develop LVNC later in life. This supports the hypothesis that LVNC may represent a morphologic continuum of genetic cardiomyopathies, including dilated and hypertrophic cardiomyopathies [14]. Sporadic occurrence is thought to account for up to 60–70% of the cases [12]. In addition, LVNC has association with Barth syndrome, mitochondrial disorders, and myotonic dystrophy [7].

*Genes Identified*. The identified genes are as follows:

 Fbkp1a/Notch pathway.
 G4.5 gene/TAZ protein.
 14-3-3 deletion.
 ZASP protein.
 TNNT2 protein.
 MYH7 protein.
 TPM1 protein.
 MYBPC3 protein.
 ACTC1 protein.

The Heart Rhythm Society states that genetic testing is recommended (Class I) for relatives and appropriate family members when a mutation-specific gene has been identified in the index case [12]. The molecular genetics of inherited cardiomyopathies have been reviewed and the evidence to support routine genetic testing in all patients diagnosed with LVNC is not available [11, 12]. The current genes available for testing are variants of established dilated cardiomyopathy and hypertrophic cardiomyopathy genes [11].

## 4. Diagnostic Approach

There is much debate regarding the diagnostic criteria for LVNC and the predilection for overdiagnosis. One case report expresses the importance of not making the diagnosis of LVNC based purely on visualized estimate on echocardiography. It is important to consider the entire diagnostic criterion as described in this section to avoid overdiagnosis [15]. In this case report, a patient was diagnosed with LVNC based on prominent trabeculations and intertrabecular recesses, but there was a better explanation for the cardiomyopathy based on the patient's history, and acute myocarditis was confirmed with cardiac magnetic resonance imaging (CMR) and myocardial biopsy.

We will review the current diagnostic criteria available for two-dimensional echocardiography, cardiac magnetic resonance imaging (CMR), and computed tomography (CT) imaging. Multiple modalities may be required for complete assessment.

### 4.1. Two-Dimensional Echocardiography

The traditional diagnostic study for evaluation of LVNC is echocardiography [1, 16–19]. It is still the most common initial test that identifies the characteristic findings of LVNC and may lead to further evaluation. There are three proposed diagnostic criteria that are most utilized in the literature. These criteria are summarized in Table 1. Chin et al. are credited with the first attempt at defining specific criteria for the diagnosis of LVNC [20]. The evaluation includes left ventricular (LV) free-wall thickness at end-diastole, prominent trabeculations, and a progressive decrease in the ratio of myocardial thickness from the epicardial surface to the trough (*X*) and the epicardial surface to the peak (*Y*) of the trabeculations in the PSAX and apical views. Stöllberger and Finsterer refined the definition as >3 trabeculations protruding from the LV wall apical to the papillary muscles, perfused intertrabecular spaces, and a two-layered myocardium with the noncompacted layer usually thicker than the compacted myocardium in end-systole [21]. We feel that the Stöllberger criteria alone may lead to the overdiagnosis of LVNC because the inclusion criteria are less detailed than other criteria and they were largely extrapolated from a large postmortem study. Subsequent recent studies have used the Jenni criteria to evaluate the presence of LVNC [13, 16]. These criteria include a bilayered myocardium, a noncompacted to compacted ratio >2 : 1, communication with the intertrabecular space demonstrated by Doppler, absence of coexisting cardiac abnormalities, and presence of multiple prominent trabeculations in end-systole [22].

Many articles have attempted to narrow the evaluation of LVNC to either end-systole or end-diastole, but a recent refinement of the echographic criteria by Stöllberger et al. stressed the importance of including both in consideration of the diagnosis [17]. In their study, three experts with more than 17 years of experience with LVNC reviewed a total of 115 echocardiograms that were proposed for inclusion in a registry. There diagnostic criteria were as follows: (1) >3 prominent trabecular formations along the left ventricular endocardial border, which are visible in end-diastole, distinct from papillary muscles, false tendons, or aberrant bands, (2) trabeculations move synchronously with the compacted myocardium, (3) trabeculations form the noncompacted part of the two-layer myocardial structure, best visible at end-systole, and (4) perfusion of the intertrabecular spaces from the ventricular cavity is present at end-diastole on color-Doppler echocardiography or contrast echocardiography. They excluded 11 patients based on their review. All experts agreed that measurement of the myocardial layers is not feasible due to the lack of uniformly accepted standards for measurements. Paterick et al. suggest measuring the myocardial layers in end-diastole based on the American Society of Echocardiography Guidelines for Chamber Quantification, which suggests wall thickness be measured in end-diastole [23]. They suggest the images for evaluation focus on nontangential short-axis views of the LV apex with special attention to the apicolateral wall. We agree on using criteria that consider both end-systolic and end-diastolic parameters, but validation of this criterion is still needed. The reproducibility of the current criteria has been tested in a small case-control study in which two echocardiography observers were blinded to the index diagnosis and reviewed 104 studies of patients with LVNC. They were in agreement with the initial diagnosis of LVNC only 67% of the time [18].

There have been recent evaluations using speckle tracking echocardiography and real-time 3-dimensional imaging (RT3DE) [24]. Speckle tracking is able to identify the abnormal ventricular mechanics by demonstrating that the basal and apical rotations are in the same direction. Real-time 3DE is useful for evaluation of LV function and quantification of trabeculations. The number of trabeculations and LV mass has been underestimated using 2-dimensional imaging when compared to RT3DE. In a study comparing 60 patients with LVNC to age-matched controls, rigid body rotation was identified in 32 of the 60 patients with LVNC [6]. This was significantly more than the normal controls and the 28 patients with normal rotation still had significantly less twist than the control patients. The patients with LVNC and rigid body rotation had worse NYHA functional status as well. The studies using both of these modalities are limited so their incorporation into the diagnosis is unclear at this time but may be considered for guidance when the diagnosis is unclear.

There are some disadvantages when using echocardiography for the assessment of LVNC. These include the inaccuracy of off-axis or oblique image planes and the challenges of evaluating the apex. These are overcome with skilled sonographers obtaining standard chamber views and the addition of contrast when the apex is not will visualized.

### 4.2. Cardiac Magnetic Resonance Imaging (CMR)

The high resolution imaging of cardiac magnetic resonance (CMR) has allowed improvement in differentiating the noncompacted and compacted myocardium. Key features of CMR in addition to the spatial resolution are the ability to image the apex well and the use of late gadolinium enhancement for the evaluation of fibrosis. A study of magnetic resonance imaging demonstrated that there are age- and sex-related differences in trabeculated and compacted myocardium of 120 normal volunteers that must be taken into consideration when making the diagnosis of LVNC [25]. They found that there is an increase in the compacted layer after the fourth decade, but a decrease in the trabecular layer. Kawel et al. analyzed the MRI findings of the 1000 participants of the Multiethnic Study of Atherosclerosis (MESA). They found that 43% of the patients without cardiac disease or hypertension had at least one of eight regions evaluated with trabeculated to compacted myocardial ratio >2.3 [26].

**Figure 1:**
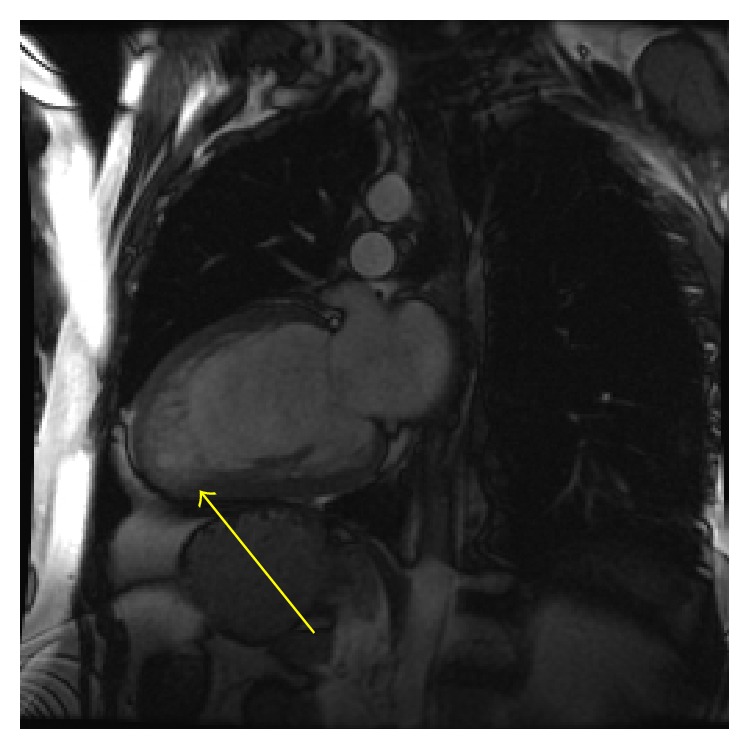
Cardiac magnetic resonance imaging (CMR) of a 38-year-old male that presented with a new cardiomyopathy and congestive heart failure. Note the prominent apical trabeculations compared to the compacted myocardium. Cardiac catheterization demonstrated no angiographic coronary disease and LVNC was the most likely diagnosis.

In 2005, Petersen et al. compared the noncompacted to compacted layers of myocardium on CMR of healthy volunteers and patients with hypertrophic cardiomyopathy, dilated cardiomyopathy, hypertensive heart disease, and aortic stenosis and patients previously diagnosed with LVNC based on other findings [27]. They found that pathological noncompaction had a NC/C >2.3 in end-diastole and that the specificity and negative predictive values were both 99%. Later in 2010, Jacquier et al. proposed that the trabeculated mass be taken into consideration when they found that the percentage of trabeculated mass was three times higher in patients with LVNC compared to other groups including controls [28]. LV trabecular mass >20% of the global mass predicted the diagnosis of LVNC with a sensitivity and specificity of 93.7%. An example of these findings is shown in Figure 1. The advantage of this later method is not depending on the evaluation of specific myocardial segments, but rather the entire mass. The patients in this study population were not provided in detail with demographic data and they note one limitation as needing to validate this method in other ethnic groups.

Dodd et al. performed a blinded, retrospective CMR review of patients with LVNC and control subjects to compare the extent and severity of noncompaction and the degree of late gadolinium enhancement (LGE) between the groups [29]. They found that the degree of delayed enhancement correlated significantly with the ejection fraction (EF). They also found that the severity of the delayed enhancement differed among patients with mild, moderate, and severe disease. This is supported in a study of patients with cardiomyopathy and progressive LV dysfunction, which correlated with a larger extent of LGE [30]. A case correspondence by Chaowu et al. correlated the late gadolinium enhancement on CMR with histopathological evidence of fibrosis in a 27-year-old patient with LVNC that underwent cardiac transplant [31]. In contrast to these findings, one study reviewed the CMR of 47 patients diagnosed with LVNC and found that the characteristics of LGE were heterogeneous [32]. Despite the nonspecific findings in the later study, the degree of LGE is significant because it is associated with the degree of LV reverse remodeling and LV dysfunction in patients with nonischemic cardiomyopathy [33].

Disadvantages of this modality include the availability of MRI and the time to complete the exam and required breath holding that pose challenges to the patients. There is also an inability to image patients with some devices/implants. We feel that CMR should play a major role in the evaluation of patients with LVNC when (1) the diagnosis by echocardiogram is not confirmed; (2) a good quality echocardiogram cannot be obtained; and/or (3) the degree of fibrosis may help delineate the severity of disease.

### 4.3. Cardiac Computed Tomography

There have been case reports in the literature that identify the diagnosis of LVNC by cardiac computed tomography (CT) [34]. The spatial resolution for identification of LVNC is good with cardiac CT [34, 35]. CT imaging also allows for visualization of the coronary arteries and great vessels. There are studies demonstrating the high specificity and negative predictive value for exclusion of CAD [36]. An advantage of using CT is the capability to evaluate the presence of both CAD and LVNC in patient with new heart failure and low likelihood of CAD. The disadvantages of CT imaging are the high radiation exposure and reactions to the contrast dye, including renal failure. The standard use of cardiac CT in evaluation of LVNC is not yet established.

## 5. Management

Management of patients with LVNC is complicated because prospective studies of large cohorts do not exist. There is limited data prospectively assessing specific agents for long-term outcomes in LVNC. Beta-blocker therapy was evaluated in a small, retrospective study of patients with LVNC in which the LV mass was reduced on beta-blocker therapy compared to the patients that were not on beta-blocker therapy at approximately one-year follow-up [37]. All patients had reduced ejection fractions at baseline, which did not show significant improvement. In general, medications for LVNC should include evidence-based, guideline directed medical therapy for patients with cardiomyopathy [38]. The standard of care for patients with dilated cardiomyopathy has been extrapolated to LVNC patients with reduced ejection fraction, but there are a few management issues that are unique to LVNC patients, including anticoagulation and primary prevention of sudden cardiac death (SCD).

### 5.1. Anticoagulation

The event rate of stroke in patients with LVNC is 1-2% per year or a total risk thromboembolism of 21–38% [39, 40]. In a review of 144 patients diagnosed with LVNC, 22 patients experienced stroke or embolism [40]. Sixty-four percent of these patients had reduced left ventricular function evaluated by echocardiogram, which suggests a higher risk of embolism in these patients. It is not clarified in the paper what is the percentage of patients with reduced EF who also had atrial fibrillation (AF), but 27% of the patients with an event also had AF. This identifies patients with reduced EF and AF as being at higher risk for stroke or embolism. Stöllberger et al. analyzed the risk of embolism or stroke in LVNC patients using the CHADS_2_/CHADS_2_-Vasc scores [41]. They found in their retrospective analysis that the patients with LVNC and a history of stroke or embolism had significantly higher CHADS_2_/CHADS_2_-Vasc scores. These patients also had a higher occurrence of atrial fibrillation, but it was not statistically significant. The CHADS_2_/CHADS_2_-Vasc score may play a role in decision-making regarding oral anticoagulation for patients with LVNC. Oral anticoagulation should be used when a definite left ventricular clot has been identified on imaging or the patient has documented atrial fibrillation. For the patients that do not fall into either of these categories, we suggest a risk assessment using the CHADS_2_/CHADS_2_-Vasc scores as guidance and a discussion with the patient regarding the risks and benefits of anticoagulation.

### 5.2. Primary Prevention of Sudden Cardiac Death

One of the greatest challenges of managing LVNC patients is whether or not an internal cardiac defibrillator (ICD) should be placed for primary prevention. Many patients with LVNC present with reduced left ventricular function, but the guidelines for patients with decreased LV function excluded patients with LVNC. In 2011, Caliskan et al. evaluated the indications and outcomes in patients with LVNC [42]. They concluded that it is appropriate to apply the current guidelines for implantation of ICD for primary or secondary prevention to patients with LVNC. The challenge is that, as previously mentioned, CMR imaging has identified late gadolinium enhancement in patients with LVNC and histopathologic confirmation of fibrosis has been performed. In the situation of normal left ventricular function with fibrosis identified on CMR the question is whether or not to place an ICD. In the previously mentioned study they required an additional risk factor for ICD implantation in patients with reduced ejection fraction (EF) and no clinical heart failure. They were family history of SCD, nonsustained VT (NSVT) on Holter monitoring, and/or self-reported history of syncope. Interestingly, 8% of the patients that received the ICD in the primary prevention group had NSVT on Holter monitoring compared to 7% of patients that received an ICD for secondary prevention of ventricular arrhythmias. This may suggest a role for applying Holter monitoring to patients with normal ejection fractions to assess the risk for SCD. One case report identified a 63-year woman with atrial tachycardia and monomorphic VT during exercise testing for evaluation of chest pain and CMR testing revealed the diagnosis of LVNC [43]. Her nuclear perfusion imaging was normal and her echocardiogram revealed only borderline left ventricular enlargement with normal left ventricular function. This supports the theory that these patients are at higher risk for SCD even with normal EF.

## 6. Summary

The overwhelming theme upon review of the current literature is that the exact method for diagnosis is still in progress, but what is clear is that the mortality of patients identified with LVNC is significant. Patients have a high incidence of NYHA III-IV heart failure, sudden cardiac death, and transplant [44–46]. It is important to take a comprehensive approach to evaluation and not rely on a single diagnostic study or parameter.

The initial study of choice remains echocardiography. Based on the current literature, we recommend the use of the Jenni criteria with consideration of both the end-diastolic and end-systolic myocardial layer thickness. As mentioned earlier, the Jenni criteria include a bilayered myocardium, a noncompacted to compacted ratio >2 : 1, communication with the intertrabecular space demonstrated by color Doppler, absence of coexisting cardiac abnormalities, and the presence of multiple prominent trabeculations in end-systole [22]. If the diagnosis is indeterminate based on the echocardiogram, then additional imaging modalities should be performed. Contrast echocardiography may be applied to further define the trabeculations and endocardium for accurate measurement of the myocardial layers. If more information is needed, then a CMR may be the reasonable next test. This will also allow for the assessment of fibrosis. If the patient is young with minimal risk for CAD and cardiac catheterization is being considered in evaluation of a new cardiomyopathy, then cardiac CT is reasonable.

Once the diagnosis has been made, then the next step is to define management goals based on the ejection fraction and degree of symptoms. If the patient meets the criteria for ICD based on reduced EF, then it is reasonable to proceed with this intervention [47]. If the patient has a normal EF, but a history of syncope, nonsustained ventricular tachycardia, or a family history of sudden cardiac death (SCD), then the clinician should review the risks and benefits of ICD therapy with the patient as an option for management. It is reasonable to take into consideration the degree of late gadolinium enhancement on MRI if this is known. For patients with reduced EF, there is evidence for the use of anticoagulation based on risk assessment with the CHADS_2_-Vasc score. If the patient has a normal EF without atrial fibrillation or visible clot, then the benefit of anticoagulation has not yet been demonstrated.

As we increase recognition and further define this cardiomyopathy it will allow for additional prospective studies to improve management and outcomes of this population.

## Figures and Tables

**Table 1:** Echocardiographic diagnostic criteria.

Criteria	Chin	Jenni	Stöllberger
Description	(i) Prominent trabeculations with deep recesses (ii) Decrease in ratio from MV level to papillary muscle level of the distance from the epicardium to the trough of the trabeculations (*X*) to the epicardium to the peak of the trabeculations (*Y*) (iii) Increasing LV wall thickness from base to apex	(i) Bilayered myocardium with multiple, prominent trabeculations in end-systole (ii) NC/C ratio of >2 : 1 (iii) Communication with the intertrabecular space demonstrated with color Doppler (iv) Absence of coexisting cardiac abnormalities	(i) Two-layer myocardium in which the noncompacted layer is thicker than the compacted myocardium (ii) >3 prominent trabeculations protruding from the LV wall apical to the papillary muscles (iii) Perfused intertrabecular spaces

Phase	End-diastole	End-systole	N/A

## References

[1] Maron B. J., Towbin J. A., Thiene G. (2006). Contemporary definitions and classification of the cardiomyopathies: an American heart association scientific statement from the council on clinical cardiology, heart failure and transplantation committee; quality of care and outcomes research and functional genomics and translational biology interdisciplinary working groups; and council on epidemiology and prevention. *Circulation*.

[2] Dusek J., Ostadal B., Duskova M. (1975). Postnatal persistence of spongy myocardium with embryonic blood supply. *Archives of Pathology*.

[3] Engberding R., Bender F. (1984). Identification of a rare congenital anomaly of the myocardium by twodimensional echocardiography: persistence of isolated myocardial sinusoids. *The American Journal of Cardiology*.

[4] Sedmera D., McQuinn T. (2008). Embryogenesis of the heart muscle. *Heart Failure Clinics*.

[5] Henderson D. J., Anderson R. H. (2009). The development and structure of the ventricles in the human heart. *Pediatric Cardiology*.

[6] Peters F., Khandheria B. K., Libhaber E. (2014). Left ventricular twist in left ventricular noncompaction. *European Heart Journal—Cardiovascular Imaging*.

[7] Finsterer J. (2009). Cardiogenetics, neurogenetics, and pathogenetics of left ventricular hypertrabeculation/noncompaction. *Pediatric Cardiology*.

[8] Probst S., Oechslin E., Schuler P. (2011). Sarcomere gene mutations in isolated left ventricular noncompaction cardiomyopathy do not predict clinical phenotype. *Circulation: Cardiovascular Genetics*.

[9] Towbin J. A. (2010). Left ventricular noncompaction: a new form of heart failure. *Heart Failure Clinics*.

[10] Hoedemaekers Y. M., Caliskan K., Michels M. (2010). The importance of genetic counseling, DNA diagnostics, and cardiologic family screening in left ventricular noncompaction cardiomyopathy. *Circulation: Cardiovascular Genetics*.

[11] Teekakirikul P., Kelly M. A., Rehm H. L., Lakdawala N. K., Funke B. H. (2013). Inherited cardiomyopathies: molecular genetics and clinical genetic testing in the postgenomic era. *The Journal of Molecular Diagnostics*.

[12] Ackerman M. J., Priori S. G., Willems S. (2011). HRS/EHRA expert consensus statement on the state of genetic testing for the channelopathies and cardiomyopathies this document was developed as a partnership between the Heart Rhythm Society (HRS) and the European Heart Rhythm Association (EHRA). *Heart Rhythm*.

[13] Bhatia N. L., Tajik A. J., Wilansky S., Steidley D. E., Mookadam F. (2011). Isolated noncompaction of the left ventricular myocardium in adults: a systematic overview. *Journal of Cardiac Failure*.

[14] Oechslin E., Jenni R. (2011). Left ventricular non-compaction revisited: a distinct phenotype with genetic heterogeneity?. *European Heart Journal*.

[15] Niemann M., Störk S., Weidemann F. (2012). Left ventricular noncompaction cardiomyopathy: an overdiagnosed disease. *Circulation*.

[16] Habib G., Charron P., Eicher J.-C. (2011). Isolated left ventricular non-compaction in adults: clinical and echocardiographic features in 105 patients. Results from a French registry. *European Journal of Heart Failure*.

[17] Stöllberger C., Gerecke B., Finsterer J., Engberding R. (2013). Refinement of echocardiographic criteria for left ventricular noncompaction. *International Journal of Cardiology*.

[18] Saleeb S. F., Margossian R., Spencer C. T. (2012). Reproducibility of echocardiographic diagnosis of left ventricular noncompaction. *Journal of the American Society of Echocardiography*.

[19] Jost C. H. A., Connolly H. M. (2012). Left ventricular non-compaction: dreaming of the perfect diagnostic tool. *European Journal of Heart Failure*.

[20] Chin T. K., Perloff J. K., Williams R. G., Jue K., Mohrmann R. (1990). Isolated noncompaction of left ventricular myocardium. A study of eight cases. *Circulation*.

[21] Stöllberger C., Finsterer J. (2004). Left ventricular hypertrabeculation/noncompaction. *Journal of the American Society of Echocardiography*.

[22] Jenni R., Oechslin E., Schneider J., Attenhofer Jost C., Kaufmann P. A. (2001). Echocardiographic and pathoanatomical characteristics of isolated left ventricular non-compaction: a step towards classification as a distinct cardiomyopathy. *Heart*.

[23] Paterick T. E., Umland M. M., Jan M. F. (2012). Left ventricular noncompaction: a 25-Year Odyssey. *Journal of the American Society of Echocardiography*.

[24] Nemes A., Kalapos A., Domsik P., Forster T. (2012). Identification of left ventricular ‘rigid body rotation’ by three-dimensional speckle-tracking echocardiography in a patient with noncompaction of the left ventricle: a case from the MAGYAR-path study. *Echocardiography*.

[25] Dawson D. K., MacEira A. M., Raj V. J., Graham C., Pennell D. J., Kilner P. J. (2011). Regional thicknesses and thickening of compacted and trabeculated myocardial layers of the normal left ventricle studied by cardiovascular magnetic resonance. *Circulation: Cardiovascular Imaging*.

[26] Kawel N., Nacif M., Arai A. E. (2012). Trabeculated (noncompacted) and compact myocardium in adults: the multi-ethnic study of atherosclerosis. *Circulation: Cardiovascular Imaging*.

[27] Petersen S. E., Selvanayagam J. B., Wiesmann F. (2005). Left ventricular non-compaction: insights from cardiovascular magnetic resonance imaging. *Journal of the American College of Cardiology*.

[28] Jacquier A., Thuny F., Jop B. (2010). Measurement of trabeculated left ventricular mass using cardiac magnetic resonance imaging in the diagnosis of left ventricular non-compaction. *European Heart Journal*.

[29] Dodd J. D., Holmvang G., Hoffmann U. (2007). Quantification of left ventricular noncompaction and trabecular delayed hyperenhancement with cardiac MRI: correlation with clinical severity. *American Journal of Roentgenology*.

[30] Masci P. G., Schuurman R., Andrea B. (2013). Myocardial fibrosis as a key determinant of left ventricular remodeling in idiopathic dilated cardiomyopathy: a contrast-enhanced cardiovascular magnetic study. *Circulation: Cardiovascular Imaging*.

[31] Chaowu Y., Li L., Shihua Z. (2011). Histopathological features of delayed enhancement cardiovascular magnetic resonance in isolated left ventricular noncompaction. *Journal of the American College of Cardiology*.

[32] Wan J., Zhao S., Cheng H. (2013). Varied distributions of late gadolinium enhancement found among patients meeting cardiovascular magnetic resonance criteria for isolated left ventricular non-compaction. *Journal of Cardiovascular Magnetic Resonance*.

[33] Kida K., Yoneyama K., Kobayashi Y., Takano M., Akashi Y. J., Miyake F. (2013). Late gadolinium enhancement on cardiac magnetic resonance images predicts reverse remodeling in patients with nonischemic cardiomyopathy treated with carvedilol. *International Journal of Cardiology*.

[34] Benjamin M. M., Khetan R. A., Kowal R. C., Schussler J. M. (2012). Diagnosis of left ventricular noncompaction by computed tomography. *Baylor University Medical Center Proceedings*.

[35] Gandhi R. T., Sarraf G., Budoff M. (2010). Isolated noncompaction of the left ventricular myocardium diagnosed upon cardiovascular multidetector computed tomography. *Texas Heart Institute Journal*.

[36] Fazel P., Peterman M. A., Schussler J. M. (2009). Three-year outcomes and cost analysis in patients receiving 64-slice computed tomographic coronary angiography for chest pain. *The American Journal of Cardiology*.

[37] Li J., Franke J., Pribe-Wolferts R. (2015). Effects of beta-blocker therapy on electrocardiographic and echocardiographic characteristics of left ventricular noncompaction. *Clinical Research in Cardiology*.

[38] Yancy C. W., Jessup M., Bozkurt B. (2013). 2013 ACCF/AHA guideline for the management of heart failure: a report of the American college of cardiology foundation/american heart association task force on practice guidelines. *Journal of the American College of Cardiology*.

[39] Cevik C., Shah N., Wilson J. M., Stainback R. F. (2012). Multiple left ventricular: in a patient with left ventricular noncompaction. *Texas Heart Institute Journal*.

[40] Stöllberger C., Blazek G., Dobias C., Hanafin A., Wegner C., Finsterer J. (2011). Frequency of stroke and embolism in left ventricular hypertrabeculation/noncompaction. *The American Journal of Cardiology*.

[41] Stöllberger C., Wegner C., Finsterer J. (2013). CHADS_2_- and CHA_2_DS_2_VASc scores and embolic risk in left ventricular hypertrabeculation/noncompaction. *Journal of Stroke and Cerebrovascular Diseases*.

[42] Caliskan K., Szili-Torok T., Theuns D. A. M. J. (2011). Indications and outcome of implantable cardioverter-defibrillators for primary and secondary prophylaxis in patients with noncompaction cardiomyopathy. *Journal of Cardiovascular Electrophysiology*.

[43] Seethala S., Knollman F., McNamara D. (2011). Exercise-induced atrial and ventricular tachycardias in a patient with left ventricular noncompaction and normal ejection fraction. *Pacing and Clinical Electrophysiology*.

[44] Tian T., Liu Y., Gao L. (2013). Isolated left ventricular noncompaction: clinical profile and prognosis in 106 adult patients. *Heart and Vessels*.

[45] Jefferies J. L., Wilkinson J. D., Sleeper L. A. (2015). Cardiomyopathy phenotypes and outcomes for children with left ventricular myocardial noncompaction: results from the pediatric cardiomyopathy registry. *Journal of Cardiac Failure*.

[46] Peters F., Khandheria B. K., Botha F. (2014). Clinical outcomes in patients with isolated left ventricular noncompaction and heart failure. *Journal of Cardiac Failure*.

[47] Yancy C. W., Jessup M., Bozkurt B. (2013). 2013 ACCF/AHA guideline for the management of heart failure: a report of the American college of cardiology foundation/American heart association task force on practice guidelines. *Journal of the American College of Cardiology*.

